# Inflammatory and anti-inflammatory markers in plasma: from late pregnancy to early postpartum

**DOI:** 10.1038/s41598-018-38304-w

**Published:** 2019-02-12

**Authors:** Emma Bränn, Åsa Edvinsson, Anna Rostedt Punga, Inger Sundström-Poromaa, Alkistis Skalkidou

**Affiliations:** 1 Department of Women’s and Children’s Health, Uppsala University, Uppsala, Sweden; 2 Department of Neuroscience, Uppsala University, Uppsala, Sweden

## Abstract

During pregnancy, the woman’s body undergoes tremendous changes in immune system adaptation. The immunological shifts that occur in pregnancy can partially be explained by alterations in hormonal levels. Furthermore, during pregnancy, many autoimmune diseases go into remission, only to flare again in the early postpartum period. Given these important changes in the clinical course of a number of autoimmune disorders, surprisingly little has been done to investigate the inflammatory profile changes across pregnancy and the postpartum period. Thus, the aim of this study was to describe how inflammatory and anti-inflammatory markers change from late pregnancy to the early postpartum period, using a multiplexed assay consisting of both well-known as well as exploratory proteins. Two-hundred-and-ninety women were included in this study and donated a total of 312 blood samples; 198 in late pregnancy (~gw38) and 114 in the postpartum period (~w8). The plasma blood samples were analyzed for 92 immune system related protein markers using Proseek Multiplex Inflammation I panel, a high-sensitivity assay based on proximity extension assay technology. Fifty-six inflammatory and anti-inflammatory markers were significantly different between pregnancy and the postpartum, of which 50 survived corrections for multiple comparisons. Out of these 50 markers, 41 decreased from pregnancy to postpartum, while the remaining 9 increased in the postpartum period. The top five markers with the greatest decrease in the postpartum period were Leukemia inhibitory factor receptor (LIF-R), Latency-associated peptide Transforming growth factor beta-1 (LAP TGF-beta-1), C-C motif chemokine 28 (CCL28), Oncostatin M (OSM) and Fibroblast growth factor 21 (FGF21). Top three markers that increased in the postpartum period were Tumor necrosis factor ligand superfamily member 11 (TRANCE), Tumor necrosis factor ligand superfamily member 12 (TWEAK), and C-C motif chemokine/Eotaxin (CCL11). This study revealed that the majority of the markers decreased from pregnancy to postpartum, and only a few increased. Several of the top proteins that were higher in pregnancy than postpartum have anti-inflammatory and immune modulatory properties promoting pregnancy progress. These results clearly reflect the tremendous change in the immune system in the pregnancy to postpartum transition.

## Introduction

During pregnancy, the woman’s body undergoes tremendous changes in immune system adaptation. At the same time as the fetus needs to be protected against pathogens, the female body needs to maintain a tolerance to paternal alloantigens in order to prevent rejection of the fetus^1^.

The immunological shifts that occur in pregnancy can partially be explained by alterations in hormonal levels, including progesterone, estradiol, and other proteins like leukemic inhibitory factor (LIF), as well as prostaglandins^2,3^. While pregnancy mainly is characterized by an anti-inflammatory immunological tolerance, inflammatory events take place during different phases of pregnancy, including implantation, placentation and in preparation for delivery. Implantation is characterized by increased levels of pro-inflammatory chemokines, cytokines and growth factors^4^. The immune system response at this stage mainly involves mast cells, dendritic cells, monocytes and macrophages. Of importance, macrophages can present in different forms, M1-macrophages and M2-macrophages, and the switch from M1- to M2- decidual macrophages is mediated by interleukin (IL)-10 and macrophage colony-stimulating factor **(**M-CSF)^5^. Thus, the first stage of pregnancy is dominated by M1-macrophages, which promote inflammation by metabolizing the amino acid arginine to nitric oxide, a molecule toxic to pathogens, and by secreting a number of pro-inflammatory cytokines such as Tumor necrosis factor (TNF)-α, IL-6 and IL-1β^4,6^.

During placental development, the previous dominance of M1-macrophages is succeeded by a more anti-inflammatory M2-milieu^7^. M2-macrophages secrete IL-10 and Transforming growth factor (TGF)-β and promote tissue-healing^8^. The M2-milieu continues into the second and third trimester with anti-inflammatory dominance, and the second trimester is characterized by rapid fetal growth and protection against preterm contractions^7^. In addition to the importance of the M1-M2 balance, the T lymphocyte profile plays an important role in the maintenance of pregnancy. Both hormonal changes and placental trophoblast immunomodulatory molecules are believed to play a role in the switch to a predominantly T helper type 2 (Th2) cell profile. Th2 and regulatory T (Treg) cells inhibit, by production of IL-4 and IL-10, the allograft rejection promoted by T helper type 1 (Th1) and T helper type 17 (Th17) cells. Moreover, Tregs are important in the maintenance of pregnancy, probably due to their production of IL-10 and TGF-β^9,10^. Further, pregnancy, with its associated hormones rising, might negatively regulate sub-populations of B cell development to avoid autoimmunity and rejection of the fetus, while enhancing antibody production in other sub-populations of B cells responsible for protection against pathogens^11^.

In preparation of delivery, another shift back to a pro-inflammatory state occurs. Cells of the immune system start migrating into the myometrium and high levels of pro-inflammatory cytokines have been found both in the cervical tissue and in the peripheral blood^12–15^. M1 macrophages in the uterus contribute to an inflammatory profile promoting uterine contractions, delivery of the baby, expulsion of the placenta, and uterine involution^16,17^.

In women, the reproductive years coincide with the period when several autoimmune disorders have their peak onset. Furthermore, during pregnancy, many autoimmune diseases go into remission, only to flare again in the early postpartum period. Typically, Th1 and Th17-type autoimmune disorders improve as a result of the rise in Th2-type cytokines in pregnancy (e.g. rheumatoid arthritis, Graves’ disease, the autoimmune neuromuscular myasthenia gravis (MG) and multiple sclerosis (MS)^18–21^), whereas Th2-type autoimmune disorders may deteriorate in pregnancy (e.g. systemic lupus erythematosus^22^). In addition, immunosuppressive regulatory B cells have been shown to increase during pregnancy, of potential relevance for the clinical course of the autoimmune disorders^23^. After delivery, the immune system returns to a non-pregnant state. A shift towards Th1 dominance, and a fall in Th2 and Treg cells, followed by altered cytokine pattern in the first weeks following delivery have been reported^1,24,25^. All of these changes may result in the worsening of Th1 and Th17-type autoimmune diseases in the postpartum period^9^.

Given these important changes in the clinical course of a number of autoimmune disorders, surprisingly little has been done to investigate the inflammatory profile changes across pregnancy and the postpartum period. For instance, previous studies have merely focused on a limited number of inflammatory and anti-inflammatory markers, primarily IL-6, IL-8 TNF-α, IL-1β and IL-10^26–29^ and a more comprehensive picture of differences in inflammatory profile in pregnancy versus postpartum is still missing. Thus, the aim of this study was to describe how inflammatory and anti-inflammatory markers change from late pregnancy to the early postpartum period, using a high-sensitivity multiplexed assay consisting of both well-known and exploratory proteins.

## Materials and Methods

### Participants

This sub-study is part of an ongoing longitudinal cohort-project, started in 2009, named the BASIC-study (Biology, Affect, Stress, Imaging and Cognition) with the aim to study peripartum depression. All Swedish speaking pregnant women over 18 years of age without confidential personal data, scheduled for a routine ultrasound at Uppsala University hospital are asked to participate in the study.

Following informed consent, information on ongoing medical and obstetric conditions, use of medication, smoking habits, pre-pregnancy body mass index and life style factors are collected by online surveys filled in by the women at gestational week 17 and 32 and at 6 weeks and 6 months postpartum. In addition to the background characteristics, the Edinburgh postnatal depression scale (EPDS) is included in all surveys.

For the purpose of the present study, we used blood samples obtained from two different sources within the BASIC framework, obtained between 2010 and 2013. First, blood samples from women, participating in a nested case-control sub-study, in gestational week 38 (median 19 (IQR 24-13) days before partus) or at 8 weeks (median 69.5 (IQR 62–77) days after partus) postpartum, or both, were used. Women invited for the sub-study comprised a selection of women with depressive symptoms according to the online EPDS survey, women on antidepressant treatment, and a corresponding number of women without depressive symptoms. Upon inclusion, the women were asked to fill in the EPDS again and were interviewed with MINI International Neuropsychiatric Interview^30^. Blood samples were drawn after at least 90 minutes fasting. The second source of blood samples were derived from women in the BASIC-study who underwent planned cesarean section (CS), in gestational week 38 (median 8 (IQR 11–5) days before ultrasound-determined date of partus), at Uppsala University hospital. In these women, venous blood samples were collected prior to surgery, following overnight fasting. Independent of source, all blood samples were centrifuged at 1500 RCF for 10 minutes and stored in −70 °C within one hour. Further, all blood samples were collected between 8:00 and 15:00, with the majority of samples drawn around 9:00 or 13:00. These conditions were similar for the samples obtained during pregnancy and postpartum.

Five hundred–and-thirty plasma samples from these two parts of the BASIC-study were analyzed, representing 482 unique women. As previous studies in our group had found profound changes in inflammatory and anti-inflammatory markers in women with antenatal depression^31^, we excluded 139 women with EPDS scores ≥13 at any time-point during pregnancy or ≥12 at any time-point during the postpartum period^32,33^. In line with this reasoning, we also excluded 33 women who were on antidepressant treatment at the time of blood sampling and 8 women with missing information on EPDS scores. Further, we excluded two duplex pregnancies, two women on oral corticosteroid treatment, one woman with preeclampsia, and finally 7 samples that could not be analyzed for technical reasons. Women with inflammatory/autoimmune disorders (celiac disease, psoriatic arthritis, ulcerative colitis and Crohn’s disease) in remission, i.e. not requiring corticosteroid treatment, were kept in the study but were adjusted for in the statistical analyses.

All in all, 290 women were included in this sub-study and donated a total of 312 blood samples; 198 in late pregnancy (129 in the case-control sub-study and 69 from the CS group) and 114 in the postpartum period. Given this, 22 women had donated samples in both pregnancy (19 in the case-control sub-study and 3 from the CS group) and postpartum.

The study procedures were in accordance with ethical standards for human experimentation and the study was approved by the Regional Ethical Review Board in Uppsala.

### Proximity extension assay

The plasma blood samples were analyzed for 92 immune system related protein markers at the Clinical Biomarker Facility at SciLifeLab Uppsala using Proseek Multiplex Inflammation I panel (Olink Bioscience, Sweden) (https://www.olink.com/products/inflammation/biomarkers/), which is based on proximity extension assay (PEA) technology^34,35^. The PEA uses two oligonucleotide-labeled antibodies for each target protein. When the two antibodies are in close proximity, a new polymerase chain reaction (PCR) target sequence is formed by proximity-dependent DNA polymerization. The resulting sequence is subsequently detected and quantified using real-time PCR.

Plasma samples were transferred to 96-well plates, each consisting of 90 samples and 6 controls (three negative controls (buffer) and three interplate controls). Each plasma sample (1 µL) was mixed with 3 µL incubation mix containing 92 antibody pairs, spiked in with two incubation controls (green fluorescent protein and phycoerythrin), one extension control and one detection control, and allowed to incubate at 4 °C overnight. Further, 96 µL extension mix, containing PEA enzyme and PCR reagents, was added, and the samples were incubated for 5 min at room temperature before the plate was transferred to the thermal cycler for an initial DNA extension at 50 °C for 20 min, followed by 17 cycles of DNA amplification. A 96.96 Dynamic Array IFC (Fluidigm, South San Francisco, CA, USA) was prepared and primed according to the manufacturer’s instructions. In a new plate, 2.8 µL of sample mixture was mixed with 7.2 µL detection mix from which 5 µL was loaded into the right side of the primed 96.96 Dynamic Array IFC.

Results were presented as Normalized protein expression (NPX), which corresponds to log2(expression), obtained in GenEx software using Olink Wizard by normalizing Cq-values against extension control, interplate control and a correction factor, and NPX corresponds to relative quantification between samples. The extension control is subtracted from the Cq-value of every sample in order to correct for technical variation and the interplate control is subtracted to compensate for possible variation between runs. Finally, the NPX is determined by normalization against a calculation correction factor that accounts for background noise.

The assay has sensitivity down to fg/mL and detects relative protein values that can be used for comparison between groups, although not for absolute quantification. Limit of detection (LOD) was determined for each biomarker based on the mean value of triplicate negative controls analyzed in each run. According to a notification from the company, brain-derived neurotrophic factor (BDNF) has been excluded from the Inflammation panel I because of a potential technical issue. For this reason, we discarded the results from the BDNF assay in our study.

### Statistics

Only markers that had NPX values higher than the limit of detection (LOD) for more than 50% of the women in pregnancy and postpartum were used in the analyses, leaving 70 markers for the statistical analyses. Included markers are presented in Supplementary Table 1, and excluded markers in Supplementary Table 2. The markers are presented with the percentage of samples with detectable protein levels. For handling of values below level of detection (LOD), the NPX values <LOD were replaced by LOD/square root(2)^36^.

Demographic data were compared using Chi-square tests, T-test and Mann-Whitney U-test as suited. Inflammatory and anti-inflammatory markers were compared between pregnancy and postpartum using Linear Mixed models, by which the cross-sectional and longitudinal design of the study was fully utilized. These analyses were adjusted for age (continuous), pre-pregnancy BMI (continuous), use of antibiotics at blood sampling (yes/no), and chronic inflammatory or rheumatic diseases (yes/no). Bonferroni correction was applied to test for multiple comparisons. The results from the Linear Mixed model were validated by Wilcoxon paired tests in the subset of 22 women who had donated a blood sample both during pregnancy and in the postpartum period.

All statistical analyses were performed using IBM SPSS statistics version 24.0. P-values below 0.05 were considered a statistical significant difference.

## Results

Demographic and clinical variables of the study population are presented in Table 1. The mean age of the women in this study was approximately 32 years, and the mean body mass index (BMI) before pregnancy was around 23 kg/m^2^. No differences were found in parity, assisted reproduction in terms of *in vitro* fertilization, smoking habits, inflammatory/rheumatoid diseases, use of antibiotics, preterm birth, or offspring gender. EPDS scores were slightly higher in the pregnancy group although within the range considered non-depressed. Among the postpartum women, 107 (93.8%) were breastfeeding. Breastfeeding status did not affect the overall results obtained from this study, data not shown.

**Table 1:** Demographic and clinical variables in the study population.

	n	Pregnancy	n	Postpartum	*p*-value^*^
Age years, mean ± SD	198	32.5 ± 4.2	114	31.9 ± 4.2	0.260
Pre-pregnancy BMI, kg/m^2^, median (IQR)	198	23.1 (21.2–25.6)	114	22.8 (21.1–25.2)	0.500
Nulliparous before this pregnancy, n (%)	198	68 (34.3)	114	56 (49.1)	0.078
*In vitro* fertilization, n (%)	169	9 (5.3)	111	8 (7.2)	0.519
Smoking, n (%)	198	5 (2.5)	114	3 (2.6)	0.954
Inflammatory/rheumatoid disease, n (%)	198	6 (3.0)	114	1 (0.9)	0.429
Use of antibiotics at blood sampling, n (%)	198	3 (1.5)	114	0 (0.0)	0.302
Preterm birth, n (%)	198	2 (1.0)	114	6 (5.3)	0.055
Gender of offspring, boy, n (%)	198	103 (52.0)	114	59 (51.8)	0.964
EPDS score, median (IQR)	198	4 (2–7)	114	2 (1–4)	<0.0001

*p-values derived from t-test, Mann-Whitney U-test and Chi^2^-test

Fifty-six inflammatory and anti-inflammatory markers were significantly different between pregnancy and the postpartum, of which 50 survived corrections for multiple comparisons (Tables 2 and 3). Out of these 50 markers, 41 were higher in pregnancy (Table 2), while the remaining 9 were higher in the postpartum period (Table 3).

**Table 2:** Markers with higher NPX values in *pregnancy*.

	Pregnancy			Postpartum			Mean difference	Linear Mixed model^*^	Linear mixed model Bonferroni *p*-value*
	n	Mean	SD	n	Mean	SD			
LIF-R	198	4.91	0.54	114	1.77	0.27	−3.14	**0.000**	**0.000**
LAP TGF-beta-1	198	7.70	0.52	114	5.29	0.35	−2.41	**0.000**	**0.000**
CCL28	198	3.71	0.89	114	1.57	0.50	−2.14	**0.000**	**0.000**
OSM	198	3.73	0.99	114	1.89	0.73	−1.84	**0.000**	**0.000**
FGF21	198	4.35	1.91	114	2.52	1.28	−1.83	**0.000**	**0.000**
HGF	198	6.87	0.46	114	5.42	0.29	−1.44	**0.000**	**0.000**
uPA	198	11.05	0.35	114	9.71	0.24	−1.34	**0.000**	**0.000**
CDCP1	198	2.75	0.56	114	1.42	0.41	−1.33	**0.000**	**0.000**
IL-10RB	198	6.27	0.39	114	4.95	0.30	−1.32	**0.000**	**0.000**
OPG (TNFRSF11)	198	10.60	0.56	114	9.30	0.30	−1.29	**0.000**	**0.000**
CSF1	198	7.91	0.29	114	6.89	0.23	−1.03	**0.000**	**0.000**
FGF23	198	2.53	1.01	114	1.54	0.48	−1.00	**0.000**	**0.000**
IL-17C	198	1.78	0.63	114	0.79	0.66	−0.98	**0.000**	**0.000**
SIRT2	198	3.81	1.36	114	2.83	1.04	−0.98	**0.000**	**0.000**
IL-18R1	198	6.44	0.49	114	5.48	0.40	−0.97	**0.000**	**0.000**
IL-18	198	7.53	0.60	114	6.59	0.49	−0.94	**0.000**	**0.000**
IL-6	198	2.33	0.75	114	1.39	0.69	−0.94	**0.000**	**0.000**
STAMBP	198	3.70	1.19	114	2.96	0.83	−0.75	**0.000**	**0.000**
CXCL10	198	8.68	0.91	114	8.04	0.78	−0.64	**0.000**	**0.000**
CXCL11	198	7.00	0.93	114	6.37	0.75	−0.63	**0.000**	**0.000**
MMP1	198	1.55	0.99	114	0.94	0.85	−0.61	**0.000**	**0.000**
4E-BP1	198	5.05	1.07	114	4.47	0.77	−0.58	**0.000**	**0.000**
AXIN1	198	2.78	1.56	114	2.21	1.21	−0.57	**0.000**	**0.007**
CASP8	198	0.69	0.75	114	0.14	0.41	−0.56	**0.000**	**0.000**
VEGF-A	198	10.18	0.33	114	9.67	0.33	−0.51	**0.000**	**0.000**
Beta-NGF	198	0.87	0.38	114	0.37	0.21	−0.49	**0.000**	**0.000**
Flt3L	198	8.54	0.43	114	8.05	0.39	−0.49	**0.000**	**0.000**
TNFSF14	198	0.98	0.60	114	0.49	0.35	−0.49	**0.000**	**0.000**
CCL3 (MIP-1-alpha)	198	1.87	0.63	114	1.38	0.39	−0.49	**0.000**	**0.000**
IL-10	198	2.65	0.75	114	2.16	0.66	−0.48	**0.000**	**0.000**
IL-7	198	2.14	0.64	114	1.65	0.48	−0.48	**0.000**	**0.000**
ADA	198	4.78	0.64	114	4.31	0.32	−0.47	**0.000**	**0.000**
CD40	198	8.38	0.64	114	7.92	0.43	−0.46	**0.000**	**0.000**
hGDNF	198	1.67	0.49	114	1.22	0.41	−0.45	**0.000**	**0.000**
IL-15RA	198	0.31	0.33	114	−0.12	0.35	−0.43	**0.000**	**0.000**
TGF-alpha	198	0.44	0.45	114	0.02	0.20	−0.42	**0.000**	**0.000**
CCL4	198	4.48	0.57	114	4.13	0.54	−0.35	**0.000**	**0.000**
DNER	198	6.46	0.42	114	6.18	0.21	−0.28	**0.000**	**0.000**
CX3CL1	198	5.11	0.49	114	4.84	0.34	−0.27	**0.000**	**0.000**
CST5	198	5.18	0.42	114	4.95	0.45	−0.22	**0.000**	**0.000**
LTA (TNFB)	198	2.87	0.53	114	2.71	0.38	−0.16	**0.000**	**0.031**
CXCL1	198	7.89	0.86	114	7.57	0.82	−0.31	**0.002**	0.123
FGF5	198	0.54	0.37	114	0.48	0.47	−0.05	**0.008**	0.569
FGF19	198	7.20	0.93	114	7.05	0.93	−0.16	0.185	
CXCL6	198	6.43	0.81	114	6.34	0.68	−0.09	0.434	
CCL25	198	5.25	0.69	114	5.17	0.54	−0.08	0.261	
CD244	198	5.24	0.47	114	5.17	0.38	−0.07	0.060	
TNFRSF9	198	5.39	0.41	114	5.36	0.32	−0.03	0.373	
IL-8 (CXCL8)	198	4.72	0.61	114	4.69	0.58	−0.03	0.796	
NT-3	198	1.71	0.73	114	1.71	0.58	−0.001	0.859	

Data are presented as number of samples, NPX mean, standard deviation (SD) and mean difference (postpartum (pp) - pregnancy (preg)), as well as Linear Mixed model- derived *p*-values and Bonferroni corrected *p*-values for the difference between postpartum and pregnancy NPX values.

^*^Adjusted for age at partus, pre-pregnancy BMI, antibiotics at blood sampling, and chronic inflammatory or rheumatic disease.

**Table 3:** Markers with higher NPX values in the *postpartum* period.

	Pregnancy			Postpartum			Mean difference	Linear Mixed model^*^	Linear mixed model Bonferroni *p*-value*
	n	Mean	SD	n	Mean	SD			
TRANCE	198	2.16	0.62	114	3.23	0.59	1.06	**0.000**	**0.000**
TWEAK	198	7.57	0.41	114	8.39	0.25	0.82	**0.000**	**0.000**
CCL11	198	6.10	0.58	114	6.88	0.51	0.77	**0.000**	**0.000**
CCL23	198	8.35	0.53	114	9.05	0.41	0.70	**0.000**	**0.000**
CXCL5	198	9.16	1.35	114	9.82	1.24	0.66	**0.000**	**0.000**
IL-12B	198	3.11	0.62	114	3.56	0.54	0.45	**0.000**	**0.000**
CCL13 (MCP4)	198	1.29	0.50	114	1.71	0.41	0.42	**0.000**	**0.000**
CD6	198	2.60	0.57	114	2.99	0.41	0.39	**0.000**	**0.000** ^**a**^
CXCL9	198	5.25	0.99	114	5.62	0.81	0.37	**0.000**	**0.018** ^**a**^
CCL20	198	5.25	0.88	114	5.45	0.82	0.20	**0.037**	2.584
CCL8 (MCP2)	198	7.57	0.75	114	7.76	0.78	0.19	**0.017**	1.210
CD5	198	3.03	0.44	114	3.15	0.27	0.12	**0.003**	0.207
TRAIL	198	7.34	0.43	114	7.45	0.24	0.11	**0.008**	0.560
S100A12 (ENRAGE)	198	0.61	0.84	114	0.72	0.48	0.11	0.163	
CCL7 (MCP3)	198	0.54	0.65	114	0.63	0.59	0.10	0.163	
CCL19	198	8.32	0.78	114	8.40	0.72	0.08	0.853	
SLAMF1	198	1.21	0.65	114	1.30	0.44	0.08	0.181	
SCF	198	7.19	0.48	114	7.23	0.30	0.05	0.456	
CCL2 (MCP1)	198	9.01	0.36	114	9.01	0.30	0.01	0.732	
SL-2 (MMP10)	198	5.33	0.70	114	5.33	0.63	0.01	0.483	

Data are presented as number of samples, NPX mean, standard deviation (SD) and mean difference (postpartum (pp) - pregnancy (preg)), as well as Linear Mixed model-derived *p*-values and Bonferroni corrected *p*-values for the difference between postpartum and pregnancy NPX values.

^*^Adjusted for age at partus, pre-pregnancy BMI, antibiotics at blood sampling, and chronic inflammatory or rheumatic disease. ^a^Not confirmed in Wilcoxon paired test.

Among the 22 participants who contributed with samples in both late pregnancy and postpartum, all but two markers (Chemokine (C-X-C motif) ligand 9 (CXCL9) and Cluster of Differentiation 6 (CD6)) were confirmed significant in the Wilcoxon test.

**Figure 1:**
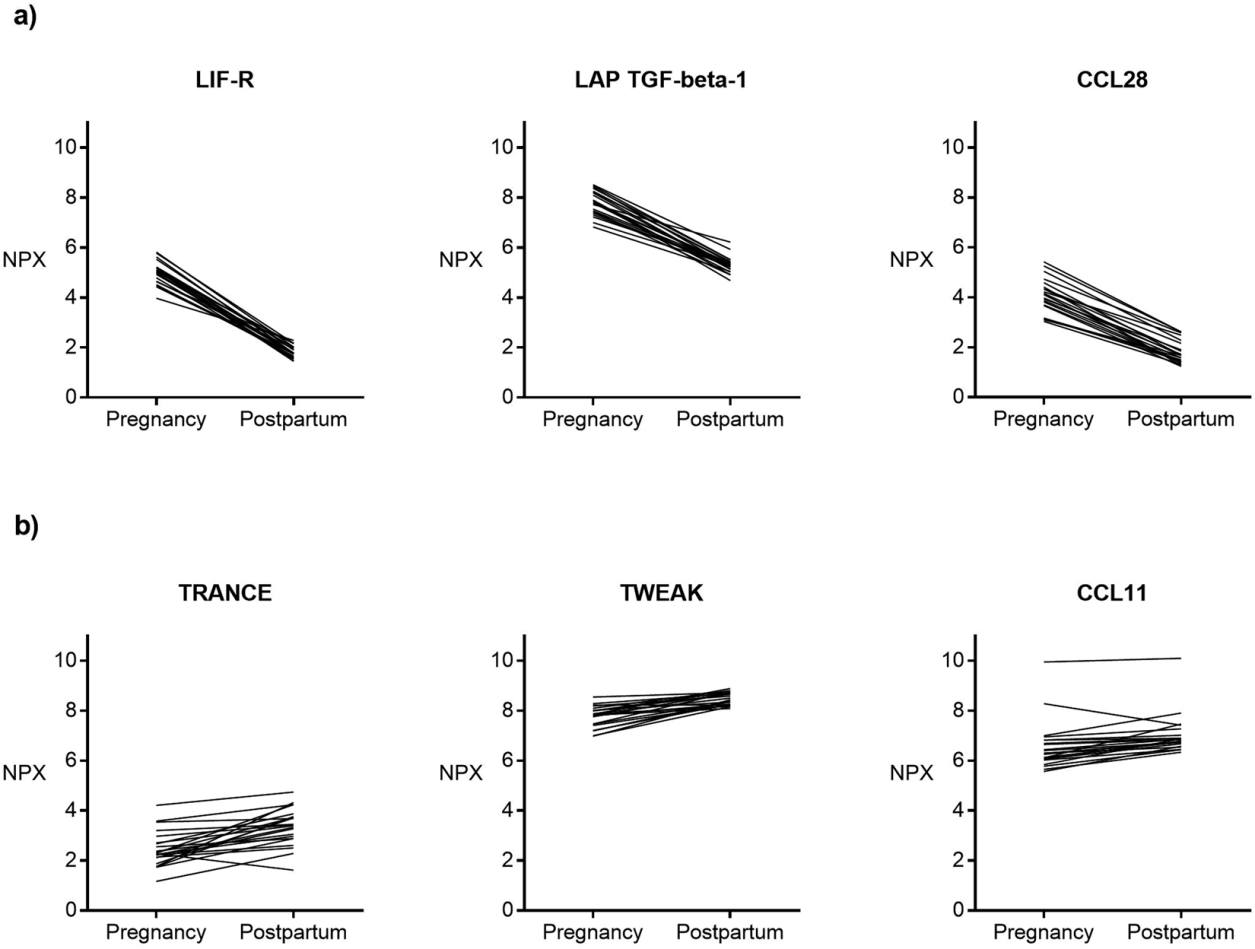
NPX values for the top markers LIF-R, LAP TGF-beta-1 and CCL28, in the individual paired samples from pregnancy to postpartum (**a**). NPX values for the top markers TRANCE, TWEAK and CCL11, in the individual paired samples from pregnancy to postpartum (**b**).

The top five markers with the greatest decrease in the postpartum period were Leukemia inhibitory factor receptor (LIF-R), Latency-associated peptide Transforming growth factor beta-1 (LAP TGF-beta-1), C-C motif chemokine 28 (CCL28), Oncostatin M (OSM) and Fibroblast growth factor 21 (FGF21) (top three markers shown in Fig. 1a). Other well-studied markers that also decrease in the postpartum period include IL-6 and IL-10.

Top three markers that increased in the postpartum period were Tumor necrosis factor ligand superfamily member 11 (TRANCE), Tumor necrosis factor ligand superfamily member 12 (TWEAK), and C-C motif chemokine/Eotaxin (CCL11) (Fig. 1b).

## Discussion

In this study we have statistically analyzed 70 inflammatory and anti-inflammatory markers in non-depressed women and found that the NPX values of 56 markers significantly differed from late pregnancy to postpartum. After correcting for multiple testing, 41 markers significantly decreased while nine markers increased in the postpartum period. The results of the paired analyses further indicate the robustness of the results. The results of this largely exploratory study on inflammatory and anti-inflammatory markers confirm, using high-sensitive proximity extension assays, the tremendous changes in immune function and inflammation status during the perinatal period. Further emphasizing the change, these decreases in inflammatory and anti-inflammatory markers were evident despite the 20% increase in plasma volume during third trimester pregnancy.

The pro- or anti-inflammatory profile of individual markers is not always easy to establish, as many of the markers have differential profiles in distinct cell-signaling pathways, tissues, or even physiological conditions^37^. Nevertheless, some typical pro-inflammatory markers are TNF-α, IL-6 and IL-1β, while IL-4, IL-10 and TGF-β on the other hand have a more prominent anti-inflammatory profile^37^. Even though the literature on inflammatory and anti-inflammatory marker changes from pregnancy to postpartum is limited, some of our results are in line with previous studies. A decrease of CXCL10 and an increase of CXCL9 from late pregnancy to early postpartum have been described previously, which is in line with our study results^38^. Decreasing levels of IL-10 from pregnancy to postpartum have been reported in an allergen-induced IL-10 production *in vitro* model^39^. A non-significant difference in levels of IL-8 has been described^27^, while another study found IL-8 to have a U-shaped curve with increasing levels postpartum^28^. The findings from previous studies of IL-6 are inconclusive. IL-6 has been reported, in line with our results, to decrease^27^, but also in contradiction to our study, to increase^28^ or to show no significant difference^26^ from pregnancy to postpartum. However, the differences in the direction of levels of inflammatory and anti-inflammatory markers from pregnancy to postpartum observed, might be due to the different sampling time points. Unfortunately, some of the markers mentioned in the literature (TNF-α and IL-1β^26–28^) were excluded from further analyses due to NPX values below LOD for more than 50% of the women in this study (IL-6), or not even included in the panel (IL-1β).

Pregnancy requires substantial alterations of multiple physiological systems in the female body, and in particular the immune system. In the postpartum period, these systems return to baseline levels. The top five markers that decreased postpartum were LIF-R, LAP TGF-beta-1, CCL28, OSM, and FGF21. LIF-R acts as a receptor for the cytokines LIF and OSM, both members of the IL-6 family^40^. LIF has anti-inflammatory properties and stimulates T regulatory cells (Tregs)^41^, and acts in a pleiotropic manner via the LIF-R on pituitary corticotrophic cells, macrophages, blastocysts, embryos, and in the placenta^42^ suggesting that higher levels of LIF-R and OSM are needed during pregnancy. LIF is described within reproduction to play an important role in implantation, embryo development, and by sufficient levels preserving the early pregnancy^42,43^. OSM seems to have both pro-inflammatory and anti-inflammatory properties^44^. OSM also possess pleiotropic actions, but seems less important within reproduction, as OSM knock-out mice appear to stay fertile^45^. OSM has been suggested to act balancing on adipogenesis/lipogenesis in peripheral organs^46^, and treatment with OSM seems to improve for example obesity and adipose tissue inflammation in mice^47^. High levels of LIF-R^48,49^ and low levels of LIF^49^ have previously been reported in pregnancy. As a whole, the LIF-R and its ligands are involved in metabolism, which may be of importance also within reproduction and preservation of an early pregnancy, which further supports the hypothesis regarding the need for higher levels of LIF-R and OSM during pregnancy than postpartum. LAP TGF-beta-1 refers to the latency-associated peptide to the TGF-beta-1 protein, that is required for several functions such as efficient secretion, prevention of binding to cell surface receptors, and extracellular availability^50,51^. Previous studies describe both LAP TGF-beta-1 and TGF-beta-1 as having immunosuppressive properties^52,53^. TGF-beta-1 has been shown to be involved in processes important in pregnancy, such as trophoblast invasion and proliferation, angiogenesis, and tolerance to the semi-allogeneic fetus^54^, suggesting even in this case a mechanism involving elevated levels during pregnancy. CCL28 (Mucosa associated epithelial chemokine (MEC)) is a chemokine that has antimicrobial and immunomodulatory properties, and is suggested to act as a bridge between the innate and the adaptive immunity^55^. Expression of CCL28 by epithelial cells, followed by an induction of pro-inflammatory cytokines/infection, helps in the recruitment of T-lymphocytes, and leads to accumulation of for example Tregs on mucosal surfaces^56^. CCL28 is suggested to have anti-inflammatory properties by limiting autoimmunity and inflammation at these sites. Elevated levels of CCL28 are also described in inflammatory diseases, such as rheumatoid arthritis^57^. CCL28 has been found to induce apoptosis in decidual stromal cells, and a higher expression of the receptors of CCL28 (CCR3, CCR10), in these cell populations in spontaneous abortion is displayed prior to a pro-inflammatory stimulation^58^. Interestingly, in mouse studies, CCL28 has been shown to be upregulated during lactation^59^, while in the current study, blood levels were lower in the postpartum period. FGF21 acts as an anti-inflammatory and immunoregulatory agent, both *in vivo* and *in vitro*^60^. Elevated plasma levels of FGF21 have been reported in pregnant women, and a positive impact of FGF21 on cardiac development in mice embryos has been described^61^. Further, FGF21 has been suggested to improve metabolism of lipids and glucose during pregnancy^62^, which could explain a need for increased levels observed during pregnancy.

The postpartum period implies extraordinary physiological processes of wound healing, lactation and sleep deprivation. The top three markers that increased postpartum were TRANCE, TWEAK, and CCL11. TRANCE is a member of the tumor necrosis factor (TNF) superfamily (TNFSF11) and can also be named RANKL^63^. TRANCE/RANKL is secreted by T cells and regulates osteoclastogenesis and bone remodeling^64^ and have been found elevated in mice induced with sleep deprivation^65^. TRANCE/RANKL secreted from human trophoblasts has also been found to polarize decidual macrophages from M1 to M2 phenotype, and decreased levels of RANKL has been noted in decidual cells from women with miscarriage^66^. Further, TRANCE is suggested to have an important role in the function of the mammary glands and for lactation in mice^67^, supporting the evidence of elevated levels of TRANCE observed postpartum. TWEAK is also a member of the tumor necrosis factor (TNF) superfamily (TNFSF12), and has been described within chronic inflammation, angiogenesis and fibrosis^68^. TWEAK is a cytokine expressed by leucocytes, monocytes, dendritic cells and natural killer cells^69^ and is suggested to be essential in tissue repair following injury^70^ which might explain the elevated levels observed in postpartum women in this study. However, in mice TWEAK is expressed by decidual NK cells, and a down-regulation of TWEAK may contribute to uterine NK cell cytotoxicity and fetal rejection^71^. CCL11 (Eotaxin-1) is a cytokine that recruits eosinophils and therefore involved in allergic responses^72^. While CCL11 has pro-invasive properties and promotes migration and invasion of trophoblasts into the uterine wall^73,74^ during pregnancy, CCL11 recruits eosinophils, neutrophils and basophiles^75^ which all play important roles in the vital tissue healing and remodeling during the postpartum period.

Notably, the two excluded markers Programmed cell death 1 ligand 1 (PD-L1) and Artemin (ARTN) switched from detectable in late pregnancy to almost undetectable in the postpartum period. This could either be due to some unknown technical issue regarding the sampling of postpartum samples, or these two markers have the most drastic decrease from pregnancy to postpartum. PD-L1 has a role in regulating T-cell homeostasis and promoting Tregs for maintenance of peripheral tolerance in pregnancy^76^. ARTN has been found in the maternal reproductive tract and embryos, suggesting its’ importance in early embryo development and pregnancy^77^.

The profound immunological alterations that occur from late pregnancy to postpartum in both pro-and anti-inflammatory proteins could play a role in the cyclic pattern of either improvement or onset/deterioration of several autoimmune diseases during pregnancy or after childbirth. In particular, Tregs are known to be defect in the autoimmune neuromuscular disorder myasthenia gravis (MG), where the risk of onset is dramatically increased in the postpartum period^78^. Even though none of the MG specific inflammatory proteins^79^ were found to be elevated in the postpartum period of healthy women, five of these proteins (TGF-α, β-NGF, IL-6, IL-17C and IL-10) were significantly higher in late pregnancy in the present study. However, the possible involvement of these inflammatory proteins in the elevated risk for postpartum onset of MG would have to be confirmed by prospective studies. Additionally, disease activity in the neuroimmune disorders MS, MG and neuromyelitis optica undergoes pronounced shifts during and after pregnancy^78,80^. The most established example is a reduction in relapse rates in the last trimester by 70–80%. Nevertheless, disease activity reappears in the first few months after delivery, temporarily overshooting pre-pregnancy levels. There are also differences in the different trimesters of pregnancy; since clinical disease activity is usually aggravated in Graves’ hyperthyroidism and MG in early pregnancy and worsening may also occur after delivery. Patients with rheumatoid arthritis experience improved clinical status during pregnancy, which is accompanied by a reduced pro-inflammatory profile^81^. Since the numbers of participants with inflammatory or rheumatic diseases in this study were very low (6 in pregnancy and 1 postpartum), we refrained from analyzing their inflammatory status separately. Thus, clarifying different autoimmune disease spectra of pro-and anti-inflammatory proteins between pregnancy and postpartum were out of the scope of the current study.

The sensitive PEA method used in combination with the large number of markers investigated, are among the strengths of this study. Cytokines are difficult to measure in the circulation due to their low concentrations. Hopefully, the highly sensitive method we used provides more robust results than previous studies, most of them being based on immunoassays. The major limitation of this study is the cross-sectional design with a low number of women followed prospectively. Additional sampling time-points could have given strengths to the study.

*In conclusion*, this study revealed 41 markers to decrease from late pregnancy to postpartum while nine markers increased. These results clearly reflect the tremendous change in the immune system in the pregnancy to postpartum transition. Several of the top proteins that were higher in pregnancy than postpartum have anti-inflammatory and immune modulatory properties promoting pregnancy progress. LIF-R and LAP TGF-beta-1, the top two markers, are well-characterized proteins within reproductive immunology and of much interest in peripartum research.

With greater understanding of the inflammatory changes the pregnant body undergoes, greater effort could be made in finding causes and treatment to pregnancy and postpartum complications and diseases thought to be related to immune function and inflammation, such as preeclampsia^82^, preterm birth^15^ and perinatal depression^26^, but even autoimmune disorders.

## Supplementary information

Supplementary table 1., Supplementary table 2.

## Data Availability

The data generated during the current study are available in the Zenodo repository 10.5281/zenodo.1249367.
