# Supplementary material for: Inflammatory and anti-inflammatory markers in plasma: from late pregnancy to early postpartum
Source: Sci Rep. 2019 Feb 12;9:1863. doi: 10.1038/s41598-018-38304-w (PMC6372606; doi:10.1038/s41598-018-38304-w)
Supplement: Supplementary file 1 — Supplementary table 1., Supplementary table 2. [file 41598_2018_38304_MOESM1_ESM.docx]

# Inflammatory and anti-inflammatory markers in plasma: from late pregnancy to early postpartum

Emma Bränn^1*^, Åsa Edvinsson^1*^, Anna Rostedt Punga^2^, Inger Sundström-Poromaa^1^, Alkistis Skalkidou^1^

^1^ Department of Women’s and Children’s Health, Uppsala University, Uppsala, Sweden

^2^ Department of Neuroscience, Uppsala University, Uppsala, Sweden

*Shared first authors, equal contribution.

Corresponding author:

Åsa Edvinsson

Department of Women´s and Children´s Health

Uppsala University

Rudbecklaboratoriet

SE- 751 85 Uppsala

Phone number: +46(0)186115772

[asa.edvinsson@kbh.uu.se](mailto:asa.edvinsson@kbh.uu.se)

**Keywords**: Pregnancy, postpartum, inflammatory markers, anti-inflammatory markers, proximity extension assay.

**Supplementary table 1**. Included proteins. Percentage of samples with detectable

levels of each protein.

|  | | Pregnancy | | Postpartum | |
| --- | --- | --- | --- | --- | --- |
| Protein | | n | Detectable protein, % | n | Detectable protein, % |
| ADA | Adenosine deaminase | 198 | 100 | 114 | 100 |
| AXIN1 | Axin-1 | 197 | 99.5 | 111 | 97.4 |
| Beta-NGF | Beta-nerve growth factor | 197 | 99.5 | 113 | 99.1 |
| CASP8 | Caspase-8 | 195 | 98.5 | 105 | 92.1 |
| CCL2 (MCP1) | C-C motif chemokine 2 (Monocyte chemotactic protein 1) | 198 | 100 | 114 | 100 |
| CCL3 (MIP-1-alpha) | C-C motif chemokine 3 (Macrophage inflammatory protein 1-alpha) | 198 | 100 | 114 | 100 |
| CCL4 | C-C motif chemokine 4 | 198 | 100 | 114 | 100 |
| CCL7 (MCP3) | C-C motif chemokine 7 (Monocyte chemotactic protein 3) | 105 | 53.0 | 77 | 67.5 |
| CCL8 (MCP2) | C-C motif chemokine 8 (Monocyte chemotactic protein 2) | 198 | 100 | 114 | 100 |
| CCL11 | C-C motif chemokine 11 (Eotaxin) | 198 | 100 | 114 | 100 |
| CCL13 (MCP4) | C-C motif chemokine 13 (Monocyte chemotactic protein 3) | 194 | 98.0 | 114 | 100 |
| CCL19 | C-C motif chemokine 19 | 198 | 100 | 114 | 100 |
| CCL20 | C-C motif chemokine 20 | 198 | 100 | 114 | 100 |
| CCL23 | C-C motif chemokine 23 | 198 | 100 | 114 | 100 |
| CCL25 | C-C motif chemokine 25 | 198 | 100 | 114 | 100 |
| CCL28 | C-C motif chemokine 28 | 198 | 100 | 114 | 100 |
| CDCP1 | CUB domain-containing protein 1 | 198 | 100 | 114 | 100 |
| CD5 | T-cell surface glycoprotein CD5 | 198 | 100 | 114 | 100 |
| CD6 | T-cell differentiation antigen CD6 | 198 | 100 | 114 | 100 |
| CD40 | B-cell surface antigen CD40  (Tumor necrosis factor receptor superfamily member 5) | 198 | 100 | 114 | 100 |
| CD244 | Natural killer cell receptor 2B4 | 198 | 100 | 114 | 100 |
| CSF1 | Macrophage colony-stimulating factor 1 | 198 | 100 | 114 | 100 |
| CST5 | Cystatin-5 (Cystatin-D) | 198 | 100 | 114 | 100 |
| CXCL1 | C-X-C motif chemokine 1 (Growth-regulated alpha protein) | 198 | 100 | 114 | 100 |
| CXCL5 | C-X-C motif chemokine 5 | 198 | 100 | 114 | 100 |
| CXCL6 | C-X-C motif chemokine 6 | 198 | 100 | 114 | 100 |
| CXCL9 | C-X-C motif chemokine 9 | 198 | 100 | 114 | 100 |
| CXCL10 | C-X-C motif chemokine 10 | 198 | 100 | 114 | 100 |
| CXCL11 | C-X-C motif chemokine 11 | 198 | 100 | 114 | 100 |
| CX3CL1 | C-X3-C motif chemokine 1 (Fractalkine) | 198 | 100 | 114 | 100 |
| DNER | Delta and Notch-like epidermal growth factor-related receptor | 198 | 100 | 114 | 100 |
| FGF5 | Fibroblast growth factor 5 | 186 | 93.9 | 111 | 97.4 |
| FGF19 | Fibroblast growth factor 19 | 198 | 100 | 114 | 100 |
| FGF21 | Fibroblast growth factor 21 | 198 | 100 | 113 | 99.1 |
| FGF23 | Fibroblast growth factor 23 | 198 | 100 | 114 | 100 |
| Flt3L | Fms-related tyrosine kinase 3 ligand | 198 | 100 | 114 | 100 |
| hGDNF | Glial cell line-derived neurotrophic factor | 174 | 87.9 | 72 | 63.2 |
| HGF | Hepatocyte growth factor | 198 | 100 | 114 | 100 |
| IL-6 | Interleukin-6 | 198 | 100 | 114 | 100 |
| IL-7 | Interleukin-6 | 198 | 100 | 114 | 100 |
| IL-8 (CXCL8) | Interleukin-8 (C-X-C motif chemokine 8) | 198 | 100 | 114 | 100 |
| IL-10 | Interleukin-10 | 198 | 100 | 114 | 100 |
| IL-10RB | Interleukin-10 receptor subunit beta | 198 | 100 | 114 | 100 |
| IL-12B | Interleukin-12 subunit beta | 198 | 100 | 114 | 100 |
| IL-15RA | Interleukin-15 receptor subunit alpha | 189 | 95.5 | 83 | 72.8 |
| IL-17C | Interleukin-17C | 197 | 99.5 | 75 | 65.8 |
| IL-18 | Interleukin-18 | 198 | 100 | 114 | 100 |
| IL-18R1 | Interleukin-18 receptor 1 | 198 | 100 | 114 | 100 |
| LAP TGF-beta-1 | [Latency-associated peptide](https://www.uniprot.org/uniprot/P01137#PRO_0000033762) transforming growth factor beta-1 | 198 | 100 | 114 | 100 |
| LIF-R | Leukemia inhibitory factor receptor | 198 | 100 | 114 | 100 |
| LTA (TNFB) | Lymphotoxin-alpha (TNF-beta) | 198 | 100 | 114 | 100 |
| MMP1 | Matrix metalloproteinase-1  (Interstitial collagenase) | 198 | 100 | 113 | 99.1 |
| NT-3 | Neurotrophin-3 | 195 | 98.5 | 113 | 99.1 |
| OPG (TNFRSF11B) | Osteoprotegerin (Tumor necrosis factor receptor superfamily member 11B) | 198 | 100 | 114 | 100 |
| OSM | Oncostatin-M | 198 | 100 | 114 | 100 |
| SCF | Stem cell factor (Kit ligand) | 198 | 100 | 114 | 100 |
| SIRT2 | NAD-dependent protein deacetylase sirtuin-2 | 198 | 100 | 114 | 100 |
| SLAMF1 | Signaling lymphocytic activation molecule | 150 | 75.8 | 102 | 89.5 |
| SL-2 (MMP10) | Stromelysin-2  (Matrix metalloproteinase-10) | 198 | 100 | 114 | 100 |
| STAMBP | STAM-binding protein | 198 | 100 | 114 | 100 |
| S100A12 (ENRAGE) | Protein S100-A12 (ENRAGE) | 178 | 89.9 | 113 | 99.1 |
| TGF-alpha | Transforming growth factor alpha | 198 | 100 | 114 | 100 |
| TNFRSF9 | Tumor necrosis factor receptor superfamily member 9 | 198 | 100 | 114 | 100 |
| TNFSF14 | Tumor necrosis factor ligand superfamily member 14 | 198 | 100 | 114 | 100 |
| TRAIL | TNF-related apoptosis-inducing ligand (Tumor necrosis factor ligand superfamily member 10) | 198 | 100 | 114 | 100 |
| TRANCE | TNF-related activation-induced cytokine (Tumor necrosis factor ligand superfamily member 11) | 198 | 100 | 114 | 100 |
| TWEAK | TNF-related weak inducer of apoptosis (Tumor necrosis factor ligand superfamily member 12) | 198 | 100 | 114 | 100 |
| uPA | Urokinase-type plasminogen activator | 198 | 100 | 114 | 100 |
| VEGF-A | Vascular endothelial growth factor A | 198 | 100 | 114 | 100 |
| 4E-BP1 | Eukaryotic translation initiation factor 4E-binding protein 1 | 198 | 100 | 114 | 100 |

**Supplementary table 2.** Excluded proteins. Percentage of samples with detectable levels of each protein.

|  | | Pregnancy | | Postpartum | |
| --- | --- | --- | --- | --- | --- |
| Protein | | n | Detectable protein, % | n | Detectable protein, % |
| ARTN | Artemin | 197 | 99.5 | 6 | 5.3 |
| IFN-gamma | Interferon gamma | 143 | 72.2 | 41 | 36.0 |
| IL-1 alpha | Interleukin-1 alpha | 13 | 6.6 | 5 | 4.4 |
| IL-2 | Interleukin-2 | 7 | 3.5 | 0 | 0 |
| IL-2RB | Interleukin-2 receptor subunit beta | 72 | 36.4 | 11 | 9.6 |
| IL-4 | Interleukin-4 | 23 | 11.6 | 14 | 12.3 |
| IL-5 | Interleukin-5 | 65 | 32.8 | 22 | 19.3 |
| IL-10RA | Interleukin-10 receptor subunit alpha | 132 | 66.7 | 49 | 43.0 |
| IL-13 | Interleukin-13 | 27 | 13.6 | 8 | 7.0 |
| IL-17A | Interleukin-17A | 90 | 45.5 | 52 | 45.6 |
| IL-20 | Interleukin-20 | 96 | 48.5 | 18 | 15.8 |
| IL-20RA | Interleukin-20 receptor subunit alpha | 41 | 20.7 | 14 | 12.3 |
| IL-22RA1 | Interleukin-22 receptor subunit alpha-1 | 21 | 10.6 | 5 | 4.4 |
| IL-24 | Interleukin-24 | 29 | 14.6 | 8 | 7.0 |
| IL-33 | Interleukin-33 | 11 | 5.6 | 1 | 0.9 |
| LIF | Leukemia inhibitory factor | 24 | 12.1 | 13 | 11.4 |
| NRTN | Neurturin | 25 | 12.6 | 12 | 10.5 |
| PD-L1 | Programmed cell death 1 ligand 1 | 198 | 100 | 6 | 5.3 |
| ST1A1 | Sulfotransferase 1A1 | 127 | 64.1 | 43 | 37.7 |
| TNF | Tumor necrosis factor | 44 | 22.2 | 16 | 14.0 |
| TSLP | Thymic stromal lymphopoietin | 14 | 7.1 | 7 | 6.1 |
